# Contribution of chronic conditions to functional limitations using a multinomial outcome: results for the older population in Belgium and Brazil

**DOI:** 10.1186/s13690-017-0235-3

**Published:** 2017-12-18

**Authors:** Renata T.C. Yokota, Wilma J. Nusselder, Jean-Marie Robine, Jean Tafforeau, Patrick Deboosere, Lenildo Moura, Silvânia S. C. A. Andrade, Shamyr S. Castro, Herman Van Oyen

**Affiliations:** 1 Department of Public Health and Surveillance, Scientific Institute of Public Health, Rue Juliette Wytsmanstraat 14, 1050 Brussels, Belgium; 2 Interface Demography, Department of Sociology, Vrije Universiteit Brussel, 1050 Brussels, Belgium; 3 Department of Public Health, Erasmus MC, Rotterdam, The Netherlands; 4 French Institute of Health and Medical Research (INSERM), Montpellier, France; 5 École Pratique des Hautes Études, Paris, France; 6 Unit for Health risks, Noncommunicable Diseases and Mental Health, Pan-American Health Organization, Brasília, Brazil; 7 Department of Noncommunicable Disease Surveillance and Health Promotion, Ministry of Health, Brasília, Brazil; 8 Department of Physiotherapy, Federal University of Ceará, Fortaleza, Brazil; 9 Department of Public Health, Ghent University, Ghent, Belgium

**Keywords:** Chronic conditions, Functional limitations, ADL, Survey, Multinomial, Brazil, Belgium

## Abstract

**Background:**

The global phenomenon of population ageing is creating new challenges in both high and middle income countries, as functional limitations are expected to increase with age. The attribution method has been proposed to identify which conditions contribute most to disability using cross-sectional data. Although the original method was based on binary outcomes, we recently proposed an extension to multinomial responses, since different disability levels are often investigated in surveys. This is the first application of the extended method to evaluate differences in the contribution of chronic conditions to functional limitations in the older population of Brazil and Belgium.

**Methods:**

Representative data from individuals aged ≥65 years who participated in the 2008 or 2013 Health Interview Surveys in Belgium (*N* = 4521) or in the 2008 National Household Sample Survey in Brazil (*N* = 28,437) were analysed. Individuals were classified as without, moderate or severe functional limitations, based on three activities of daily living: eating, showering, and toileting. Six chronic conditions common to the surveys – diabetes, heart diseases, musculoskeletal conditions, depression, chronic respiratory diseases, and cancer – were included in the analysis. Separate multinomial additive hazards models by gender for each country were fitted.

**Results:**

The prevalence of moderate functional limitations was larger in men in Brazil (8.4%) compared to Belgium (6.0%) and similar in women (approximately 12.0%). Conversely, the severe prevalence in men was similar in the two countries (around 8.0%) and higher in women from Belgium (16.6%) than from Brazil (9.1%). Musculoskeletal conditions were the main contributors to the prevalence of functional limitations in men and women in Belgium but only in men and women with moderate functional limitations in Brazil. Depression and heart diseases contributed most to the severe prevalence of functional limitations in men and women in Brazil, respectively.

**Conclusions:**

Our findings provide a better understanding of differences in the prevalence of different levels of functional limitations in Brazil and Belgium. These differences can be related to differences in socioeconomic conditions, health care access and quality, disease diagnosis, stage of epidemiology transition, life expectancy, and the prevalence of lifestyle risk factors in the two countries.

**Electronic supplementary material:**

The online version of this article (10.1186/s13690-017-0235-3) contains supplementary material, which is available to authorized users.

## Background

Although population ageing is currently considered a global phenomenon, the burden of chronic conditions and functional limitations, common at older ages, have different impacts in high and middle income countries [1, 2]. While in low and middle income countries the increase in the proportion of older individuals is related to the fast reduction of fertility rates and infant mortality, especially from infectious diseases, in high income countries this is currently a result of the decrease in mortality at older ages [2, 3]. One key difference in the increased life expectancy observed in developed and developing countries is the pace in which this process is developing: whilst it started more than a century ago in most developed countries, it is a recent phenomenon in developing countries [4]. This rapid ageing process poses challenges in developing countries as they did not grow wealth before growing old [5]. As a consequence, health care systems of less developed countries are less prepared to provide appropriate long-term care to older individuals [6, 7].

Limitations in activity of daily living (ADL) can represent difficulty or dependence in self-care [8], indicating that older individuals with functional limitations are a heterogeneous group. The assessment of different levels of functional limitations is fundamental to better understand differences in the needs and demands of older individuals, as dependency levels are associated with higher demand for long term care and mortality [9, 10].

Information on how much different chronic conditions contribute to different levels of functional limitations is crucial to develop public health strategies aiming to reduce function loss. This can be assessed with the attribution method [11, 12], in which cross-sectional data are used to estimate the prevalence of functional limitations. The prevalence of functional limitations is then partitioned into the additive contribution of diseases, taking into account that individuals can report more than one disease (multimorbidity) and that functional limitations can occur in the absence of disease. The attribution method was originally developed for binary outcomes [11, 12], but it was recently extended to allow multicategory responses [13]. Although the original attribution method has been previously used to investigate the disability burden in Belgium [14–18] and in Brazil [19] using binary outcomes, this is the first study to compare the contributions in a high and middle income country using a multinomial outcome for functional limitations, representing different severity levels.

Thus, the aim of this study was to assess the main contributors to moderate and severe functional limitations in the older population from Belgium and in Brazil.

## Methods

### Surveys and study populations

This study focused on Belgium and Brazil due to the data availability of independent, population-based, national surveys with similar questions about functional limitations and chronic conditions. The prevalence of risk factors for chronic conditions and the risk of premature mortality due to selected chronic conditions (cancer, diabetes, cardiovascular diseases, and chronic respiratory diseases) in both countries were very similar to the aggregated estimates of high and upper-middle income countries (Additional file 1), suggesting that these two countries are good representatives of the latter groups.

Data from the following national health surveys from Belgium and Brazil were used: (i) Health Interview Surveys conducted in Belgium (HISBe) in 2008 and 2013; and (ii) National Household Sample Survey conducted in Brazil (NHSSBr) in 2008. A summary of the main characteristics of each survey is presented in Table 1. Although the surveys allowed proxy interviews, the NHSSBr excluded individuals living in nursing homes or homes for the elderly. The response rate in the Brazilian survey (95%) was greater than in the Belgian surveys (2008 = 55%; 2013 = 57%). The HISBe data are available upon request and approval to the Belgian Privacy Commission <https://his.wiv-isp.be/SitePages/Acces_microdata.aspx> and the NHSSBr is openly available at <http://www.ibge.gov.br/home/estatistica/populacao/trabalhoerendimento/pnad2011/microdados.shtm>. More details about the HISBe [20] and the NHSSBr [21] methodologies can be found elsewhere.

**Table 1:** – Characteristics of the Health Interview Surveys, Belgium, 2008 and 2013 and National Household Sample Survey, Brazil, 2008

Characteristic	Belgium		Brazil
Survey year	2008	2013	2008
Sample size	11,254	10,829	391,868
Respondents aged ≥65 years with complete data	2503	2018	28,437
Response rate	55%	57%	95%
Sampling frame	National Population Register		2000 census
Sampling design	Multistage with stratification (regions and provinces), and clustering (municipality and household)		Multistage with simple random sampling (self-representative municipalities), stratification (non-representative municipalities and census tracts), and clustering (households)
Target population	Belgian population, including individuals living in nursing homes or homes for the elderly		Brazilian population
Excluded subgroups	Homeless and illegal immigrants not included in the sampling frame and individuals living in prisons or religious communities with more than 8 residents		Military bases, prisons, homes for the elderly, homes for the children, monasteries, and hospitals
Proxy interview	Mandatory for individuals aged <15 years; allowed for individuals with severe mental or physical conditions not able to respond to the interview and individuals who refused to participate, but allowed proxy answers		Allowed
Question for chronic conditions	During the past 12 months, have you had any of the following diseases or conditions?		Has a doctor or health professional said that you have (disease/condition)?
Question for ADL limitations	Do you usually have difficulty in doing any of these activities by yourself? (Activities included in this analysis: feeding yourself, bathing or showering, and using toilets)		In general, because of a health problem, do you have difficulty to eat, take a shower, or go to the toilet?
Response options for ADL limitations questions	1. No difficulty 2. Yes, some difficulty 3. Yes, a lot of difficulty 4. I can’t achieve it by myself		1. Unable 2. A lot of difficulty 3. Some difficulty 4. No difficulty
Interview method for ADL limitations and chronic condition questions	Face-to-face interview		Face-to-face interview

ADL limitations: activities of daily living limitations

Here, we focused on the population aged 65 years or older, as functional limitations and chronic conditions are more frequent at older ages [15]. For Belgium, the data of the 2008 and 2013 surveys were pooled due to the small sample size of the 2008 HISBe, which did not allow fitting the multinomial model stratified by age and gender. Nonetheless, the prevalence of moderate and severe functional limitations was similar in the two Belgian surveys for most age groups (Additional file 2).

In total, 4521 (HISBe) and 28,437 (NHSSBr) individuals were included in this study, respectively. For comparability purposes, the questions used to define functional limitations and the chronic conditions included in this analysis were common to the surveys in both countries.

Functional limitations were defined based on limitations in three activities of daily living (ADL): eating, showering, and toileting (Table 1). These three ADLs cover the whole ADL spectrum: eating is considered the easiest task, which is generally lost late in life; toileting is an intermediate task, with middle loss; and bathing is a difficult task, with an early loss [22–24]. The multicategory outcome was defined as “0. No functional limitations”, for individuals who answered “No difficulty”; “1. Moderate functional limitations”, for individuals who responded “Some difficulty”; and “2. Severe functional limitations”, for individuals who answered “A lot of difficulty” or “Unable” in the NHSSBr or “A lot of difficulty” or “Can’t achieve it by myself” in the HISBe. The last response options were grouped due to sparseness.

Six chronic conditions were considered in the present analysis: diabetes, heart diseases (myocardial infarction and coronary heart diseases), chronic respiratory diseases (HISBe: asthma, chronic bronchitis, pulmonary emphysema, chronic obstructive pulmonary diseases (COPD); NHSSBr: asthma and chronic bronchitis); musculoskeletal conditions (arthritis and back pain), depression, and cancer.

### Statistical analysis

To improve comparability between the two countries, age and gender standardized disease prevalence is presented, based on direct standardization [25], with the world population used as standard.

The attribution method [11, 12] was used to estimate the contribution of chronic conditions to the prevalence of functional limitations, taking into account multimorbidity and that functional limitations can be present even in the absence of diseases. In individuals with functional limitations who did not report any of the considered diseases, functional limitations are attributed to “background”, while in individuals who reported diseases, functional limitations are partitioned into “background” and diseases. The background can represent the effect of age-related losses in functioning, functional limitations that are not associated with any disease (e.g. the environment), causes of functional limitations not included in the survey, and underreported or underdiagnosed diseases. More information about the attribution method can be found in previous publications [11, 12].

Here, the extended attribution method was used, allowing multicategory responses. This extension is based on the multinomial additive hazard model [13], defined as:

1 $$ {\displaystyle \begin{array}{l}y\sim Multinomial\left(1,\overset{\wedge }{y_j}\right)\\ {}\overset{\wedge }{y_j}=\left[1-\exp \left(-\sum \limits_{j=2}^c{\eta}_q\right)\right]\left(\frac{\eta_j}{\sum_{j=2}^c{\eta}_q}\right)\\ {}{\eta}_j={\alpha}_{aj}+\sum \limits_{d=1}^m{\beta}_{adj}{X}_a{X}_d\end{array}} $$

where *y*(1, …, *j*) is the observed multicategory outcome for functional limitations, with *y*_1_ defined as the reference category (no functional limitations), *y*_2_ as moderate functional limitations and *y*_3_ as severe functional limitations; $\overset{\wedge }{y_j}$ is the estimated probability for functional limitation level *j*; *η*_*j*_ is the linear predictor representing the overall cumulative hazard of functional limitation level *j*; *α*_*aj*_ is the cumulative hazard of functional limitation level *j* for background by age group *a*(65–74 years; ≥75 years); *β*_*adj*_ is the cumulative hazard of functional limitation level *j* for disease *d*(1, …, *m*) and age group *a*, also known as the disabling impact of disease [11, 12]; *X*_*a*_ is the indicator variable for each age group *a*; and *X*_*d*_ is the indicator variable for each disease *d*. To estimate the model parameters (*α*_*aj*_ and *β*_*adj*_) constrained optimization [26] was used.

In the model above, the attribution of functional limitations to diseases depends on the disease prevalence by age group (*X*_*a*_ *X*_*d*_) and the disabling impacts of the diseases (*β*_*adj*_) [11, 12]. The contribution of diseases and background to the moderate and severe prevalence of functional limitations was obtained in three steps [13]:

1. Similar to the competing risks analysis, the proportionality assumption [27] was used to estimate the disease-specific probability of moderate (*j* = 2) and severe (*j* = 3) functional limitations, ${\hat{D}}_{dj}=\frac{\beta_{adj}{X}_a{X}_d}{\eta_j}\times {\hat{y}}_j$, and for the background, ${\hat{B}}_j=\frac{\alpha_{aj}}{\eta_j}\times {\hat{y}}_j$, for each individual;
2. Next, the number of individuals with moderate (*j* = 2) and severe (*j* = 3) functional limitations by each disease, ${\hat{N}}_{dj}={\sum}_{i=1}^n{\hat{D}}_{dj}$, and by background, ${\hat{N}}_{bj}={\sum}_{i=1}^n\hat{B_j}$, are obtained by the sum of cause-specific probabilities in the population studied;
3. The absolute contribution to the moderate (*j* = 2) and severe (*j* = 3) prevalence of functional limitations (${\hat{AC}}_j$) of each disease (${\hat{AC}}_{dj}=\frac{{\hat{N}}_{dj}}{n}\times 100$) and background (${\hat{AC}}_{bj}=\frac{{\hat{N}}_{bj}}{n}\times 100$), i.e. the prevalence of moderate and severe functional limitations by cause, is estimated by dividing the total number of individuals with moderate and severe functional limitations for each cause by the total number of individuals in the population studied.

The absolute contributions and the moderate and severe prevalence of functional limitations for Belgium and Brazil were age-standardized, using direct standardization [25] and the world population as standard. The confidence intervals for the disease prevalence and the disabling impacts were obtained via bootstrapping [28]. All the analysis were carried out in R [29]. The multinomial additive hazard model was fitted with the R package “addhaz” [30].

## Results

Table 2 shows the characteristics of the study population of each country. Although the age distribution of individuals without functional limitations is similar between the countries, a higher proportion of older individuals is observed in the Belgian population with moderate and severe functional limitations compared to Brazil. The proportion of lower educated men and women in Brazil was much larger than in Belgium across all subgroups. The proportion of men and women who did not report any selected chronic conditions was similar among men and women without functional limitations in the two countries, but higher in Brazilian men and women with moderate and severe functional limitations compared to Belgium. The proportion of multimorbidity (≥2 selected chronic conditions) was similar in the male population from Belgium and Brazil and higher in women from Brazil than in women from Belgium.

**Table 2:** Characteristics of the study population. Health Interview Surveys, Belgium, 2008 and 2013 and National Household Sample Survey, Brazil, 2008

Characteristic	Men											
	Belgium						Brazil					
	No limitations		Moderate		Severe		No limitations		Moderate		Severe	
	N	%^a^	N	%^a^	N	%^a^	N	%^a^	N	%^a^	N	%^a^
Age group (years)												
65–69	465	33.8	19	17.5	15	8.0	4143	39.5	233	23.5	206	21.8
70–74	351	27.7	15	12.2	21	15.5	2879	28.6	234	24.2	179	18.5
75–79	291	19.7	28	19.3	45	19.7	1803	17.7	202	21.9	200	21.3
80–84	206	13.5	40	33.0	43	27.2	914	8.8	144	15.0	168	18.9
≥ 85	206	5.3	63	18.0	132	29.6	543	5.3	145	15.3	185	19.5
Education level												
No diploma/primary	407	26.8	55	36.6	113	50.8	7943	77.7	830	87.5	786	84.6
Secondary	678	47.9	74	45.9	93	35.9	750	6.9	53	5.0	45	4.8
Tertiary	407	24.2	32	16.4	38	12.2	1589	15.4	75	7.5	107	10.6
Missing information	27	1.5	4	1.1	12	1.1	0	0	0	0	0	0
Number of chronic conditions^b^												
0	671	44.7	48	21.2	51	20.2	4635	44.8	245	26.4	235	26.1
1	547	36.5	58	35.0	96	33.1	3796	36.9	376	39.2	321	34.0
≥ 2	263	16.1	50	40.3	95	42.9	1851	18.3	337	34.4	382	39.9
Missing information	38	2.7	9	3.5	14	3.9	0	0	0	0	0	0
	Women											
Age group (years)												
65–69	481	29.9	38	14.4	18	5.3	4977	38.3	416	23.1	224	15.1
70–74	389	27.7	42	17.9	48	10.2	3487	27.1	427	23.1	248	16.4
75–79	338	19.9	56	23.2	83	16.1	2363	18.3	395	21.7	303	19.0
80–84	260	15.2	66	24.0	149	31.7	1327	10.4	283	16.7	328	21.8
≥ 85	334	7.3	137	20.4	467	36.7	759	5.9	281	15.4	441	27.7
Education level												
No diploma/primary	590	31.7	139	44.3	392	58.3	10,196	79.6	1582	88.4	1362	88.7
Secondary	853	51.1	144	42.7	241	30.8	988	7.6	93	4.9	76	4.6
Tertiary	328	16.0	43	11.6	48	5.0	1720	12.6	126	6.7	106	6.7
Missing information	31	1.2	13	1.4	84	5.8	9	0.1	1	0.1	0	0
Number of chronic conditions^b^												
0	664	36.7	77	18.8	151	15.7	4466	34.8	372	21.8	310	19.9
1	744	41.2	142	40.7	307	40.3	5079	38.7	691	37.8	517	32.4
≥ 2	338	19.3	99	36.4	261	38.5	3368	26.5	739	40.4	717	47.7
Missing information	56	2.8	21	4.1	46	5.6	0	0	0	0	0	0

^a^Weighted prevalence is presented

^b^Chronic conditions considered: diabetes, heart diseases, chronic respiratory diseases, musculoskeletal conditions, depression, and cancer

**Fig. 1:**
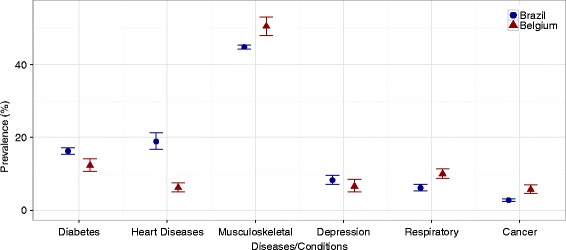
– Age and gender standardized disease prevalence. Health Interview Surveys, Belgium, 2008 and 2013 and National Household Sample Survey, Brazil, 2008. Heart diseases: myocardial infarction and coronary heart disease.Musculoskeletal: arthritis and back pain. Respiratory: asthma and chronic bronchitis (Brazil); and asthma, chronic bronchitis, pulmonary emphysema, chronic obstructive pulmonary diseases (Belgium). Direct standardization used with the world population as standard

Musculoskeletal conditions (back pain and arthritis) were the most common chronic conditions in Belgium and Brazil, while cancer was the least frequent condition in both countries (Fig. 1). The prevalence of musculoskeletal conditions (Brazil: 44.8%, 95% CI: 44.3%–45.4%; Belgium: 50.5%, 95% CI: 48.0%–53.0%), chronic respiratory conditions (Brazil: 6.2%, 95% CI: 5.4%–7.1%; Belgium: 10.0%, 95% CI: 8.8%–11.4%), and cancer (Brazil: 2.7%, 95% CI: 2.4%–3.2%; Belgium: 5.7%, 95% CI: 4.6%–7.0%) was higher in Belgium than in Brazil, whilst the prevalence of diabetes (Brazil: 16.3%, 95% CI: 15.4%; 17.2%; Belgium: 12.3%, 95% CI: 10.8%; 14.1%) and heart diseases (Brazil: 18.9%, 95% CI: 16.7%; 21.3%; Belgium: 6.2%, 95% CI: 5.1%; 7.5%) was higher in Brazil than in Belgium (Fig. 1).

Tables 3 and 4 show the disabling impacts and the background cumulative hazards for men and women, respectively. In Belgium, depression and cancer were the most disabling diseases in men aged 65–74 years and 75 years or older with moderate functional limitations, respectively, whereas chronic respiratory diseases showed the highest impact in men with severe functional limitations. In Brazil, depression was the most disabling condition for men with moderate and severe functional limitations across all age groups (Table 3).

**Table 3:** Background cumulative hazard and rank of the disabling impact by age group for men. Health Interview Surveys, Belgium, 2008 and 2013 and National Household Sample Survey, Brazil, 2008

	Belgium											
Rank	Moderate						Severe					
	65–74 years			≥75 years			65–74 years			≥75 years		
	Condition	DI	CI	Condition	DI	CI	Condition	DI	CI	Condition	DI	CI
1	Depression	0.19	−0.01; 0.65	Cancer	0.15	−0.04; 0.45	Respiratory	0.12	0.02; 2.82	Respiratory	0.17	0.02; 0.36
2	Respiratory	0.08	0.00; 0.20	Depression	0.07	−0.07; 0.43	Depression	0.12	−0.18; 4.42	Musculoskeletal	0.14	0.06; 0.23
3	Diabetes	0.04	−0.01; 0.10	Musculoskeletal	0.06	0.00; 0.12	Cancer	0.03	−0.33; 0.60	Diabetes	0.10	−0.01; 0.26
4	Musculoskeletal	0.01	−0.01; 0.03	Diabetes	0.05	−0.05; 0.18	Heart diseases	0.02	−0.48; 1.24	Depression	0.10	−0.07; 0.51
5	Cancer	0.01	−0.01; 0.12	Heart diseases	0.04	−0.04; 0.15	Musculoskeletal	0.00	−0.03; 0.04	Heart diseases	0.09	−0.05; 0.25
6	Heart diseases	0.00	−0.02; 0.11	Respiratory	0.04	−0.05; 0.16	Diabetes	0.00	−3.46; 0.07	Cancer	0.00	−0.12; 0.18
–	Background	0.01	0.00; 0.02	Background	0.06	0.04; 0.10	Background	0.02	0.00; 0.05	Background	0.08	0.05; 0.12
	Brazil											
Rank	Moderate						Severe					
	65–74 years			≥75 years			65–74 years			≥75 years		
	Condition	DI	CI	Condition	DI	CI	Condition	DI	CI	Condition	DI	CI
1	Depression	0.10	0.06; 0.15	Background	0.09	0.08; 0.11	Depression	0.10	0.06; 0.15	Depression	0.33	0.22; 0.46
2	Cancer	0.07	0.02; 0.11	Depression	0.07	0.00; 0.14	Cancer	0.07	0.02; 0.12	Cancer	0.17	0.08; 0.27
3	Musculoskeletal	0.04	0.03; 0.05	Musculoskeletal	0.06	0.03; 0.09	Diabetes	0.03	0.01; 0.05	Diabetes	0.09	0.05; 0.14
4	Heart diseases	0.03	0.01; 0.05	Heart diseases	0.05	0.02; 0.08	Heart diseases	0.03	0.02; 0.05	Respiratory	0.09	0.03; 0.17
5	Respiratory	0.03	0.00; 0.07	Respiratory	0.04	−0.02; 0.10	Respiratory	0.02	−0.01; 0.05	Heart diseases	0.06	0.03; 0.10
6	Diabetes	0.01	0.00; 0.03	Diabetes	0.02	−0.02; 0.06	Musculoskeletal	0.01	0.00; 0.02	Musculoskeletal	0.03	0.00; 0.05
–	Background	0.03	0.03; 0.04	Background	0.09	0.08; 0.11	Background	0.03	0.02; 0.04	Background	0.08	0.07; 0.10

Heart diseases: myocardial infarction and coronary heart disease

Musculoskeletal: arthritis and back pain

Respiratory: asthma and chronic bronchitis (Brazil); and asthma, chronic bronchitis, pulmonary emphysema, and chronic obstructive pulmonary diseases (Belgium)

**Table 4:** Background cumulative hazard and rank of the disabling impact by age group for women. Health Interview Surveys, Belgium, 2008 and 2013 and National Household Sample Survey, Brazil, 2008

	Belgium											
Rank	Moderate						Severe					
	65–74 years			≥75 years			65–74 years			≥75 years		
	Condition	DI	CI	Condition	DI	CI	Condition	DI	CI	Condition	DI	CI
1	Depression	0.27	0.00; 0.61	Depression	0.14	−0.05; 0.37	Cancer	0.09	0.03; 0.15	Depression	0.73	0.42; 1.12
2	Heart diseases	0.26	−0.01; 0.75	Musculoskeletal	0.10	0.02; 0.17	Depression	0.08	0.05; 0.11	Diabetes	0.28	0.06; 0.49
3	Musculoskeletal	0.05	0.02; 0.09	Diabetes	0.08	−0.04; 0.19	Diabetes	0.03	0.02; 0.05	Heart diseases	0.18	−0.08; 0.51
4	Cancer	0.02	−0.05; 0.11	Heart diseases	0.08	−0.09; 0.30	Heart diseases	0.03	0.02; 0.05	Musculoskeletal	0.14	0.08; 0.28
5	Respiratory	0.01	−0.04; 0.09	Respiratory	0.00	−0.13; 0.10	Respiratory	0.01	−0.01; 0.03	Cancer	0.06	−0.28; 0.53
6	Diabetes	0.00	−0.05; 0.06	Cancer	0.00	−0.13; 0.20	Musculoskeletal	0.01	0.00; 0.02	Respiratory	0.00	−0.18; 0.18
–	Background	0.04	0.02; 0.06	Background	0.11	0.08; 0.16	Background	0.03	0.02; 0.03	Background	0.26	0.18; 0.30
	Brazil											
Rank	Moderate						Severe					
	65–74 years			≥75 years			65–74 years			≥75 years		
	Condition	DI	CI	Condition	DI	CI	Condition	DI	CI	Condition	DI	CI
1	Cancer	0.06	0.00; 0.12	Heart diseases	0.07	0.03; 0.10	Cancer	0.09	0.03; 0.15	Depression	0.15	0.10; 0.21
2	Heart diseases	0.05	0.03; 0.08	Diabetes	0.06	0.01; 0.09	Depression	0.08	0.05; 0.11	Cancer	0.15	0.05; 0.27
3	Depression	0.05	0.03; 0.08	Musculoskeletal	0.05	0.03; 0.08	Diabetes	0.03	0.02; 0.05	Heart diseases	0.12	0.09; 0.16
4	Diabetes	0.03	0.01; 0.05	Depression	0.05	−0.01; 0.09	Heart diseases	0.03	0.02; 0.05	Diabetes	0.06	0.02; 0.10
5	Respiratory	0.03	0.00; 0.06	Respiratory	0.00	−0.05; 0.05	Respiratory	0.01	−0.01; 0.03	Respiratory	0.06	0.00; 0.12
6	Musculoskeletal	0.03	0.01; 0.04	Cancer	0.00	−0.07; 0.08	Musculoskeletal	0.01	0.00; 0.02	Musculoskeletal	0.02	0.00; 0.05
–	Background	0.05	0.05; 0.06	Background	0.13	0.11; 0.14	Background	0.03	0.02; 0.03	Background	0.12	0.11; 0.14

Heart diseases: myocardial infarction and coronary heart disease

Musculoskeletal: arthritis and back pain

Respiratory: asthma and chronic bronchitis (Brazil); and asthma, chronic bronchitis, pulmonary emphysema, and chronic obstructive pulmonary diseases (Belgium)

Depression was the most disabling condition for Belgian women with moderate functional limitations, whilst cancer and depression showed the highest impact on severe functional limitations in women aged 65–74 years and ≥75 years, respectively.

In Brazil, cancer was the most disabling disease for women aged 65–74 years with moderate or severe functional limitations, whereas heart diseases and depression showed the highest impact in women aged 75 years or older with moderate and severe functional limitations, respectively (Table 4).

**Fig. 2:**
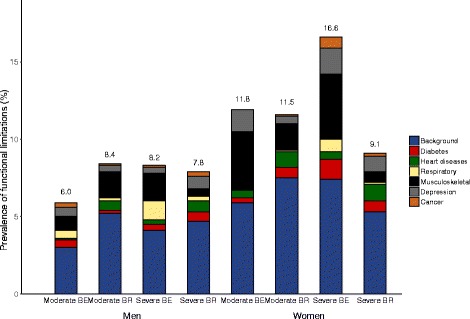
– Age-standardized prevalence of functional limitations and absolute contribution of chronic conditions and background to moderate and severe functional limitations. Health Interview Surveys, Belgium, 2008 and 2013 and National Household Sample Survey, Brazil, 2008. Heart diseases: myocardial infarction and coronary heart disease. Respiratory: asthma and chronic bronchitis (Brazil); and asthma, chronic bronchitis, pulmonary emphysema, and chronic obstructive pulmonary diseases (Belgium). The numbers on the top of the bars present the age-standardized prevalence of functional limitations, using direct standardization with the world population as the standard

The age-standardized prevalence of functional limitations and absolute contribution of chronic conditions to moderate and severe functional limitations in Belgium and Brazil is shown in Fig. 2 and in Additional file 3. Both moderate and severe functional limitations were more common in women compared to men in both countries (Fig. 2). While the moderate prevalence of functional limitations was larger in men from Brazil (8.4%) compared to Belgium (6.0%), it was similar in women from both countries (Brazil: 11.5%; Belgium: 11.8%). The severe prevalence was similar in men (Belgium: 8.2%; Brazil: 7.8%), but much larger in Belgian (16.6%) women compared to Brazilian (9.1%) women.

Musculoskeletal conditions were the main contributors to moderate functional limitations in men and women from both countries and for severe functional limitations in Belgium. Depression and heart diseases were the main contributors to the prevalence of severe functional limitations in men and women from Brazil, respectively.

## Discussion

This is the first study to compare the contribution of chronic conditions to moderate and severe functional limitations in a high and a middle income country, using the extended attribution method to multinomial disability outcomes. Although musculoskeletal conditions were among the main contributors to the moderate and severe prevalence of functional limitations in Belgium, this was observed only for moderate functional limitations in Brazil. For severe functional limitations, depression and heart diseases contributed most in Brazil.

A smaller gender gap in the prevalence of functional limitations was observed in Brazil than in Belgium, especially for severe functional limitations, indicating that in Brazil the proportion of older women (age ≥ 65 years) reporting severe difficulties/inability in feeding, bathing/showering, or using the toilet is only slightly higher than in men, while in Belgium this gender difference is more pronounced. Although the explanations for the differences between the two countries are not clear, it can reflect: (i) differences in the question formulation in the surveys; (ii) gender differences in the prevalence of chronic conditions; and (iii) cultural differences in the interpretation in the ADL questions and in reporting ADL limitations in the surveys [31]. A small gender difference in the prevalence of severe ADL limitations has been previously reported in a study with data of one Brazilian city [32].

Despite all the differences previously mentioned, a remarkable finding is that the prevalence of moderate (men and women) and severe (men) functional limitations is rather similar in Belgium and Brazil. These results seem contradictory, especially because of the differences in the ageing process in Brazil and Belgium. The current older adults in Brazil are exposed to the burden of chronic conditions and, at the same time, are survivors of infectious diseases and worse overall living conditions in their early life [33]. Besides the cultural differences in reporting functional limitations, the similarity in the prevalence of functional limitations in the two countries can be related to a mortality selection during childhood in the older population in Brazil [33]. This means that the current older individuals in Brazil were highly selected in terms of health. This is supported by the similarity in the proportion of healthy life years (very good, good or fair self-perceived health) at age 65 in Belgium (men: 86%; women: 78%) [34] and at age 60 in Brazil (men: 87%; women: 86%) [35] in 2013.

Conversely, a larger prevalence of severe functional limitations was observed in women from Belgium compared to Brazil. One possible explanation is the fact that the older population living in institutions was included in the survey in Belgium but not in Brazil. In Belgium, 6.6% of the individuals aged 65 years or older were living in long-term care institutions in 2004 [36]. Most of these individuals are women (78.5% in 2007) [37] and have ADL limitations, as this is an admission requirement [36]. Nonetheless, only 0.5% of the older individuals were living in institutions in Brazil in 2011 [38].

Musculoskeletal conditions are the most common cause of functional limitations [39] and they have been previously identified as the main contributor to the disability burden using the attribution method with different indicators in Belgium [15, 16, 18] and other European countries [40–42]. This was also found in a previous study using the HISBe data from 1997, 2001, 2004, and 2008 that investigated the main contributors to mild and severe disability burden with the attribution method using a binary outcome based on six ADL and mobility limitations, except in men with severe limitations, in which chronic respiratory diseases were identified as the main contributor [14]. These results are in agreement with our current findings: chronic respiratory diseases were the second contributor to the severe prevalence of functional limitations in Belgian men. This can be related to life style risk factors, such as tobacco smoking, which are more common among older men in Belgium [43] and to past working conditions, as approximately 10% of male labour force was related to the Belgian coal industry [44].

In Brazil, musculoskeletal conditions were the main contributors to moderate functional limitations, whereas depression and heart diseases contributed most to the severe prevalence. The attribution method has also been previously applied to the Brazilian National Health Survey data from 2013, where stroke was the main contributor in men and diabetes in women [19]. Although heart diseases were among the main contributors in women, depression was not even significant in the model and it was excluded from the analysis [19]. The differences in the main contributors compared to the present study can be due to: (i) different disability definition, as the previous study defined disability based on ADL and instrumental ADL limitations; (ii) no distinction between severity levels in the previous study, thus the results might have been driven by moderate limitations, as it is more frequent than severe disability; (iii) no adjustment for age in the previous study, hence the results for the younger age groups (65–74 years) may be overrepresented, as a higher proportion individuals aged 65–74 years was observed in the sample studied; and (iv) stroke was not included in the present analysis as it was not available in the NHSSBr in 2008 and its contribution was, therefore, captured by the background.

It is interesting to note that chronic respiratory diseases were among the main contributors to the severe prevalence of functional limitations among men and women in Belgium, but not in Brazil. Despite the different definition of chronic respiratory diseases in both surveys, as in Belgium COPD, which is an important cause of functional limitations [45], was also included in this disease group, another possible explanation is the lower prevalence of tobacco smoking observed in Brazil (men: 21.6%; women: 13.1%) [46] compared to Belgium (men: 28.0%; women: 21%) [43] among individuals aged 15 years or older in 2008. Differences in passive smoking and air pollution between the two countries may also have contributed. In the previous study with the 2013 Brazilian data, chronic respiratory diseases, which included COPD, were not important contributors to the prevalence of ADL and IADL limitations [19].

In the attribution method, the contribution of chronic diseases to the prevalence of functional limitations depends on the disease prevalence and the disabling impact of the disease [11, 12]. For instance, musculoskeletal conditions were important contributors to the prevalence of moderate functional limitations in both countries mainly due to their high prevalence in the populations, while depression and heart diseases were the main contributors to severe functional limitations in Brazil owing to their high disabling impact and prevalence, respectively. Cancer was among the most disabling diseases in Brazil, but due to its low prevalence, it was not an important contributor to the prevalence of functional limitations. The lower prevalence of cancer in Brazil compared to Belgium can be related to delayed diagnosis, resulting in shorter survival in Brazil [47].

Differences in the contributors to the prevalence of functional limitations in Belgium and Brazil can be related to dissimilarities between the countries related to: (i) health care access; (ii) quality of diagnosis and health care; (iii) socioeconomic conditions; (iv) stage of epidemiologic transition; (v) life expectancy; and (vi) prevalence of life style risk factors. For instance, a large difference in the education attainment between the study populations of the two countries was observed: in Brazil, more than 80% of the individuals with moderate or severe functional limitations reported no diploma or primary education while this proportion was around 35–60% in Belgium. In a previous study with the 1997 HISBe, individuals with low education attainment showed a higher prevalence and disabling impact for heart diseases and stroke compared to highly educated individuals [18].

Depression was an important contributor to the severe prevalence of functional limitations in Brazil. Although functional limitations were found to be short-term outcomes of depression, which can be partly explained by reduced physical activity and social participation in individuals with depression [48, 49], this relationship should be carefully interpreted, as our results are based on cross-sectional data. In other words, the causal relationship between functional limitations and depression cannot be established, which may have resulted in an overestimation of the contribution of depression to the prevalence of moderate and severe functional limitations. This temporal bias is specially observed for depression, as an inverse causal association has been previously reported: functional limitations as a predictor of onset and persistence of depression [49].

The main limitation of the attribution method is related to the use of cross-sectional data. Despite the plausibility of the causal assumption between chronic conditions and functional limitations, it cannot be assessed with cross-sectional data. This may result in incorrect attribution of disability to diseases in cases where disability occurred before diseases [11, 12]. Although longitudinal studies are better suited to assess the causal relationship between diseases and functional limitations [11], no representative longitudinal data are currently available in both countries.

The role of disease co-occurrence, which could be captured by including two-way interactions between diseases in the multinomial model, was not investigated due to the limited sample size in the Belgian surveys. The effect of disease combinations has been previously investigated in Belgium with a binary disability outcome [16], indicating that the co-occurrence of (i) chronic respiratory diseases and depression and (ii) cardiovascular diseases and diabetes were very disabling (highest disabling impacts) but not important contributors to the disability prevalence due to their low occurrence in the older population in Belgium.

The use of self-reported chronic conditions can hamper comparability between the two countries, as it is associated with health care access and medical diagnosis. Also, cultural differences may have an impact in the interpretation and the reply to questions, although most of the questions were very similar in both surveys. An overestimation of the background contribution might have occurred due to: (i) the lack of information about important causes of functional limitations such as stroke and dementia, as they were not systematically included in both surveys; and (ii) underdiagnosed diseases, especially in Brazil, as medical diagnose of diseases is related to health care access. This is supported by the higher proportion of individuals without any reported disease observed in the Brazilian population with moderate and severe functional limitations.

Although previous studies with binary disability outcomes have shown that the contribution of diseases to the disability prevalence differ according to educational level, with higher disabling impacts observed among lower educated groups [18, 50], we were not able to account for it in our analysis, due to the limited sample size of the Belgian surveys.

Furthermore, our definition of functional limitations included only three ADLs (eating, showering, and toileting), as they were common to the surveys in both countries. Nonetheless, these three ADLs cover the whole ADL spectrum, including easy, intermediate and difficult tasks [22–24]. Finally, the use of the pooled data of the 2008 and 2013 surveys in Belgium was necessary due to the small sample size of the 2008 survey, which did not allow us to fit the multinomial model. Although this might have impacted the comparability between the results of the two countries, we expect that this impact was minimal, as the prevalence of moderate and severe functional limitations did not show substantial changes between 2008 and 2013 in Belgium (Additional file 2).

The main added value of this study was the possibility to investigate the contributors to different severity levels of functional limitations in a high and a middle income country, using the population-based survey data from both countries. Despite the small differences in the formulation of the questions in the two independent surveys, the similarity of the surveys allowed us to compare the results of the two countries.

## Conclusions

Our study showed important differences in the main contributors to the moderate and severe prevalence of functional limitations in Belgium and Brazil, which can be related to differences in disease diagnosis, health care access and quality, socioeconomic conditions, stage of epidemiology transition, life expectancy, and prevalence of risk factors for chronic conditions between the two countries. These differences should be taken into account when comparing the main contributors to the prevalence of functional limitations between developing and developed countries and should also be considered by policymakers to reduce functional limitations. For instance, in Belgium strategies focusing on both prevention and treatment of musculoskeletal conditions to delay disease progression should be targeted, as they were associated with both moderate and severe functional limitations mainly due their high occurrence in the Belgian older population. In Brazil, besides musculoskeletal conditions, which were also associated with moderate functional limitations, depression and heart diseases should be prioritized, as these diseases contributed most to severe functional limitations. Therefore, strategies to reduce severe functional limitations in Brazil should focus on prevention of heart diseases, which presented a high prevalence, and on the disease management and modification of the environment to make it more accessible for individuals with depression, as it showed a high disabling impact.

Distinguishing between severity levels in functional limitations is crucial for policymakers, as severe levels are associated with higher dependence, long-term care, social burden, and increased health care costs. Although the assessment of different severity levels of functional limitations is more informative, it should be preferred over a binary outcome when the sample size is large enough, especially when the prevalence of functional limitations is low. Future studies should take into account education attainment when investigating the differences in the prevalence of functional limitations between countries. It can also be interesting to extend the attribution method to longitudinal data, as causality could be directly assessed, keeping in mind that the model can become more complex, as several transition rates (e.g between no, moderate, and severe functional limitations) will need to be estimated.

## Additional files

Additional file 1: Risk of premature mortality due to selected chronic diseases and risk factors for chronic diseases in high income countries, upper-middle income countries, Belgium, and Brazil. (DOC 34 kb)

Additional file 2: Prevalence of moderate and severe functional limitations. Health Interview Surveys, 2008 and 2013, Belgium. (DOC 201 kb)

Additional file 3: Age-standardized prevalence of functional limitations and absolute contribution of chronic conditions and background to moderate and severe functional limitations. Health Interview Surveys, Belgium, 2008 and 2013 and National Household Sample Survey, Brazil, 2008. (DOC 33 kb)

## Abbreviations

ADL: Activity of daily living
COPD: Chronic obstructive pulmonary diseases
HISBe: Health Interview Surveys in Belgium
NHSSBr: National Household Sample Survey in Brazil
