# Supplementary material for: Contribution of chronic conditions to functional limitations using a multinomial outcome: results for the older population in Belgium and Brazil
Source: Arch Public Health. 2017 Dec 18;75:68. doi: 10.1186/s13690-017-0235-3 (PMC5733874; doi:10.1186/s13690-017-0235-3)
Supplement: Supplementary file 1 — Risk of premature mortality due to selected chronic diseases and risk factors for chronic diseases in high income countries, upper-middle income countries, Belgium, and Brazil. (DOC 34 kb) [file 13690_2017_235_MOESM1_ESM.doc]

Additional file 1 – Risk of premature mortality due to selected chronic diseases and risk factors for chronic diseases in high income countries, upper-middle income countries, Belgium, and Brazil.

| Indicator | High income countries | Belgium | Upper-middle income countries | Brazil |
| --- | --- | --- | --- | --- |
| Risk of premature mortality (30-70 years) from cancer, diabetes, CVD, or CRD)^a^ | 14.2 | 13.6 | 22.2 | 21.2 |
| Risk factors^b^ |  |  |  |  |
| Daily cigarette smoking (%)^c^ | 21.0 | 21.2 | 24.0 | 13.4 |
| Insufficient physical activity (%)^d^ | 43.0 | 42.7 | 41.0 | 49.4 |
| Alcohol consumption (litres per capita) ^e^ | 11.0 | 10.4 | 9.0 | 10.1 |
| High blood pressure (%)^f^ | 35.0 | 34.8 | 41.0 | 42.3 |
| Overweight (%)^g^ | 54.0 | 51.5 | 59.0 | 52.8 |
| Obesity (%)^h^ | 21.0 | 19.1 | 25.0 | 19.5 |

^a^Estimates from 2005. Available at<<http://apps.who.int/gho/data/node.main.A857?lang=en>>. CVD : cardiovascular diseases ; CRD : chronic respiratory diseases.

^b^Estimates from 2008. World Health Organization. Global status report on noncommunicable diseases 2010, 2011, Geneva. Available at< <http://www.who.int/nmh/publications/ncd_report_full_en.pdf>>.

^c^Age-standardized prevalence of current daily cigarette smoking in individuals aged 15 years or older.

^d^Age-standardized prevalence of physical inactivity (< 5 times, 30 minutes of moderate activity per week; or < 3 times, 20 minutes of vigorous activity per week; or equivalent) in individuals aged 15 years or older.

^e^Adult per capita consumption of alcohol (litres of pure alcohol).

^f^Age-standardized prevalence of raised blood pressure (systolic blood pressure ≥140mmHg and/or diastolic blood pressure ≥90mmHg or on medication) in individuals aged 25 years or older.

^g^Age-standardized prevalence of overweight (body mass index ≥25kg/m^2^) in individuals aged 20 years or older.

^h^Age-standardized prevalence of obesity (body mass index ≥30kg/m^2^) in individuals aged 20 years or older.
