# Supplementary figures and images for: Contribution of chronic conditions to functional limitations using a multinomial outcome: results for the older population in Belgium and Brazil
Source: Arch Public Health. 2017 Dec 18;75:68. doi: 10.1186/s13690-017-0235-3 (PMC5733874; doi:10.1186/s13690-017-0235-3)

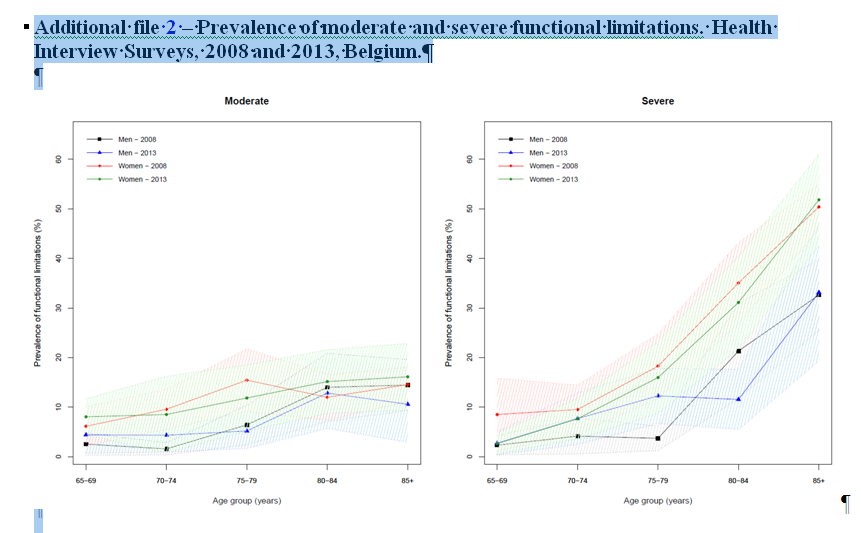

Supplement: Supplementary file 2 — Prevalence of moderate and severe functional limitations. Health Interview Surveys, 2008 and 2013, Belgium. (DOC 201 kb) [file 13690_2017_235_MOESM2_ESM.doc]
