# Supplementary material for: Contribution of chronic conditions to functional limitations using a multinomial outcome: results for the older population in Belgium and Brazil
Source: Arch Public Health. 2017 Dec 18;75:68. doi: 10.1186/s13690-017-0235-3 (PMC5733874; doi:10.1186/s13690-017-0235-3)
Supplement: Supplementary file 3 — Age-standardized prevalence of functional limitations and absolute contribution of chronic conditions and background to moderate and severe functional limitations. Health Interview Surveys, Belgium, 2008 and 2013 and National Household Sample Survey, Brazil, 2008. (DOC 33 kb) [file 13690_2017_235_MOESM3_ESM.doc]

### Additional file 3 – Age-standardized prevalence of functional limitations and absolute contribution of chronic conditions and background to moderate and severe functional limitations. Health Interview Survey, Belgium, 2008 and 2013 and National Household Sample Survey, Brazil, 2008.

| Condition | Mild | | | | Severe | | | |
| --- | --- | --- | --- | --- | --- | --- | --- | --- |
|  | Men | | Women | | Men | | Women | |
|  | Brazil | Belgium | Brazil | Belgium | Brazil | Belgium | Brazil | Belgium |
| Diabetes | 0.2 | 0.5 | 0.7 | 0.3 | 0.6 | 0.4 | 0.7 | 1.3 |
| Heart diseases | 0.6 | 0.1 | 1.0 | 0.6 | 0.7 | 0.3 | 1.1 | 0.5 |
| Respiratory diseases | 0.2 | 0.5 | 0.1 | 0.0 | 0.3 | 1.2 | 0.1 | 0.8 |
| Musculoskeletal conditions | 1.7 | 0.9 | 1.7 | 3.8 | 0.5 | 1.8 | 0.7 | 4.2 |
| Depression | 0.4 | 0.6 | 0.5 | 1.4 | 0.8 | 0.4 | 1.0 | 1.7 |
| Cancer | 0.1 | 0.3 | 0.1 | 0.0 | 0.3 | 0.1 | 0.2 | 0.7 |
| Background | 5.2 | 3.0 | 7.5 | 5.9 | 4.7 | 4.1 | 5.3 | 7.4 |
| Total prevalence of functional limitations | 8.4 | 6.0 | 11.5 | 11.8 | 7.8 | 8.2 | 9.1 | 16.6 |

Heart diseases: myocardial infarction and coronary heart disease.

Musculoskeletal conditions: arthritis and back pain.

Respiratory diseases: asthma and chronic bronchitis (Brazil); and asthma, chronic bronchitis, pulmonary emphysema, chronic obstructive pulmonary diseases (Belgium).
